# Probiotic Potential and Genome-Based Characterization of *Lactiplantibacillus plantarum* M2, a Promising Isolate Obtained from Spontaneous Fermentation of *Humiria balsamifera* Pulp

**DOI:** 10.3390/pharmaceutics17121557

**Published:** 2025-12-03

**Authors:** Carlos Drielson da Silva Pereira, Roberval Nascimento Moraes Neto, Carlos Eduardo Morais de Sousa, Enio Ciro Dantas de Farias Rocha, Diogo Zeque Bastos, Suana Millen Bruzaca Mota, Romulo Maia Ferreira, Adrielle Zagminan, Luís Cláudio Nascimento da Silva

**Affiliations:** 1 Biodiversity and Biotechnology Graduate Program (Bionorte Network), CEUMA University, São Luis 65075-120, Brazil; drielsonn.sousa@gmail.com (C.D.d.S.P.); robervalmoraes11@gmail.com (R.N.M.N.); adrielle004602@ceuma.com.br (A.Z.); 2 Laboratory of Microbial Pathogenesis, CEUMA University, São Luis 65075-120, Brazil; ceduardoak21@gmail.com (C.E.M.d.S.); eniocirodantas@gmail.com (E.C.D.d.F.R.); diogozeque@gmail.com (D.Z.B.); 3 Instituto Barão de Lucena, São Luis 65075-200, Brazil; suanabruzaca21@gmail.com; 4 LACMAR Laboratory, São Luis 65061-010, Brazil; romulo.ferreira5@hotmail.com

**Keywords:** lactic acid bacteria, probiotics, Amazonian fruits, phylogenetic analysis

## Abstract

**Background/Objectives:** The growing demand for functional foods and alternative therapeutic strategies has intensified the search for novel probiotic strains from underexplored ecosystems. This study aimed to isolate and phenotypically characterize lactic acid bacteria (LAB) from spontaneously fermented fruits found in the Legal Amazon (*Ananas comosus*, *Humiria balsamifera*, *Manilkara zapota*, and *Platonia insignis*) and to perform genome-based analysis of the most promising isolate to evaluate its probiotic potential. **Methods:** The isolates were identified by MALDI-TOF-MS and screened for tolerance to low pH, bile salts, lysozyme, growth at 39 °C, and antimicrobial activity against five enteric pathogens. The most promising isolate was evaluated by coaggregation and biofilm assays, in silico proteome and CAZyme analysis, bacteriocin cluster mining, and in vivo efficacy testing using *Tenebrio molitor* larvae. **Results:** Three isolates from *H. balsamifera* were identified as *Lactiplantibacillus plantarum* (M1, M2, M4) by MALDI-TOF-MS. These isolates exhibited high resilience to all tested physiological stressors. Antimicrobial activity was contact-dependent, with no inhibition by cell-free supernatants. M2 showed the strongest pathogen exclusion, moderate biofilm formation, and high coaggregation with *S. enterica* and *E. faecalis*. Genome analysis of M2 revealed a 3.40 Mb chromosome, absence of acquired resistance or virulence genes, two plantaricin gene clusters, and 93 CAZymes, including GT families linked to exopolysaccharides biosynthesis. SignalP predicted secretion signals in 10 CAZymes. M2 significantly improved larval survival against *E. coli* and *S. enterica*, especially under prophylactic treatment. **Conclusions:** *L. plantarum* M2 combines safety, stress tolerance, genomic features, and in vivo efficacy, positioning it as a promising probiotic candidate adapted to tropical niches. These findings highlight *H. balsamifera* as a reservoir of novel probiotic strains.

## 1. Introduction

Probiotics are defined as live microorganisms that confer health benefits to the host when administered in adequate amounts [1,2]. They promote protection against gastrointestinal pathogens and help prevent conditions such as irritable bowel syndrome and lactose intolerance while contributing to the maintenance of intestinal microbiota homeostasis. In addition to these effects, certain probiotic strains exhibit anti-inflammatory and immunomodulatory properties, whereas others may exert pro-inflammatory activity, stimulating host immune responses against pathogens [3,4]. Although various species of fungi and bacteria are used as probiotics, lactic acid bacteria (LAB) remain the most widely employed due to their safety profile and functional versatility [5,6].

The growing demand for functional foods and alternative therapeutic strategies has driven the search for novel probiotic strains with beneficial health activities [6,7,8]. Traditionally, probiotic bioprospecting has focused on sources such as fermented dairy products and human specimens (e.g., feces, vaginal samples) [6,9,10,11]. However, the biodiversity of underexplored ecosystems, such as the native vegetation of the Legal Amazon region (which encompasses the states of Acre, Amapá, Amazonas, Mato Grosso, Pará, Rondônia, Roraima, Tocantins, and Maranhão), represents a vast and promising reservoir for the discovery of microorganisms with unique functional traits and novel biotechnological applications [8,12]. In this context, the Legal Amazon region stands out as a hotspot for microbial bioprospecting, offering a rich diversity of native and regional fruits that harbor complex and potentially beneficial microbial communities.

Most commercial probiotics originate from dairy products or human sources, which may limit their ecological adaptability and functional diversity, particularly when applied to novel food matrices and advanced delivery systems [13,14]. In contrast, strains adapted to plant-based and tropical niches exhibit unique resilience profiles that are essential for stability in pharmaceutical formulations (capsules, powders) and survival during gastrointestinal transit [15]. Tropical ecosystems, such as those found in the Legal Amazon, impose environmental stresses, including temperature fluctuations, which, combined with physicochemical changes (pH, osmolarity, sugar and acid composition) occurring during fruit maturation, shape the microbiota of native fruits and favor isolates with enhanced stress tolerance and adhesion capabilities. These characteristics are critical for competitive pathogen exclusion and, consequently, for the development of effective and stable microbial therapeutics [16,17,18]. Thus, strains of plant origin are positioned as high-value therapeutic candidates, applicable in gut health interventions, infection control, and the development of advanced probiotic delivery systems [19].

Therefore, this study primarily aimed to isolate and phenotypically characterize LAB from spontaneously fermented fruits found in the Legal Amazon region: *Ananas comosus* (pineapple), *Humiria balsamifera* (mirim), *Manilkara zapota* (sapodilla), and *Platonia insignis* (bacuri). The selection of these tropical fruits was based on their ecological relevance, traditional use in local communities, and physicochemical characteristics (such as low pH, high sugar content, and rich profiles of organic acids and phenolic compounds) that favor microbial colonization and fermentation. The second phase of this study aimed to perform genome-based analysis of the most promising isolate to evaluate its probiotic potential. We hypothesized that LAB isolates adapted to tropical fruit niches exhibit superior stress tolerance and/or distinct mechanisms of pathogen antagonism, representing a relevant contribution to the field of plant-derived probiotics.

## 2. Materials and Methods

### 2.1. Sample Collection

The fruits were obtained in local markets of Humberto de Campos (*H. balsamifera*) and São Luís (*A. comusus*, *M. zapota*, *P. insignis*), two cities in Maranhão State. As a criterion, only ripe or ripening fruits showing no visible signs of phytopathology were selected. The fruits were stored in sterile self-sealing plastic bags, kept at 4 °C, and analyzed within 48 h.

### 2.2. Spontaneous Fermentation and Isolation of Lactic Acid Bacteria

The selected fruits were washed with sterile distilled water and processed immediately afterward. The samples were manually crushed, and the pulps (250 g) were placed inside sterile self-sealing plastic bags within a laminar flow cabinet. The headspace air was partially evacuated before sealing to reduce oxygen and create semi-anaerobic conditions for spontaneous fermentation and subsequent isolation of fermentative microorganisms. The samples were incubated at 25 °C for 15 days. After spontaneous fermentation, 25 g of each sample were homogenized in 225 mL of sterile peptone water. Serial dilutions ranging from 10^−2^ to 10^−5^ were prepared, and 100 µL of each dilution was plated on MRS agar (Man, Rogosa, and Sharpe; Kasvi, Pinhais, Brazil) [20]. The plates were incubated at 37 °C for 48 to 72 h under anaerobic conditions to promote the growth of LAB. Anaerobiosis was achieved using steel wool immersed in an acidified copper sulfate solution (500 mL distilled water, 1.64 mL sulfuric acid, 1 mL Tween 80, and 25 g copper sulfate), combined with CO_2_ generation through an effervescent tablet (sodium bicarbonate and citric acid) (adapted from [21]). Colonies were initially screened using Gram staining and catalase testing. Isolates that were Gram-positive and catalase-negative were selected for further identification.

### 2.3. Identification by MALDI-TOF MS Analysis

Colonies were initially screened using Gram staining and catalase testing. Isolates that were Gram-positive and catalase-negative were selected for further identification. Yeast isolates were preliminarily classified based on morphology and catalase reaction. Definitive identification of both lactic acid bacteria and yeast isolates was performed using a MALDI-TOF MS (Matrix-Assisted Laser Desorption/Ionization Time-of-Flight mass spectrometry system) with the MALDI Biotyper platform (version 4.1.100.10, Bruker Daltonics, Bremen, Germany). Briefly, fresh colonies were spotted onto a polished steel target plate, treated with 1 µL of 100% formic acid for protein extraction, and overlaid with 1 µL of α-cyano-4-hydroxycinnamic acid (HCCA) matrix. After air-drying, the plate was analyzed in linear positive mode, calibrated using the Bruker Bacterial Test Standard, which includes *Escherichia coli* DH5α as a reference strain. Spectra were processed using MALDI Biotyper software (version 4.1.100.10) and compared against the MSP Library (BDAL, revision 13), which contains reference profiles for Gram-positive and Gram-negative bacteria as well as yeast species. Identification scores ≥2.0 were considered reliable at the species level according to the manufacturer criteria.

### 2.4. Antimicrobial Susceptibility Testing of Lactiplantibacillus plantarum Isolates

The antimicrobial susceptibility of the *Lactiplantibacillus plantarum* isolates was evaluated using the disc diffusion method, following the guidelines established by the Clinical and Laboratory Standards Institute (CLSI, 2022). A bacterial suspension with a concentration of 1 × 10^8^ CFU/mL was prepared and uniformly spread onto solid MRS agar plates using sterile swabs. Antibiotic-impregnated discs were placed on the inoculated plates and incubated at 37 °C for 24–48 h. After incubation, inhibition zones were measured with a digital caliper. Isolates were classified as resistant (R), intermediate (I), or sensitive (S) according to CLSI interpretive criteria (Table S1). To validate the test conditions, standard reference strains *Escherichia coli* ATCC 25922 and *Staphylococcus aureus* ATCC 27853 were used as quality controls.

### 2.5. Evaluation of Physiological Tolerance of Lactiplantibacillus plantarum Isolates

#### 2.5.1. Thermal Tolerance

The isolates were cultured at two temperatures: 37 °C (optimal growth) and 39 °C (elevated temperature) [22]. Growth at 39 °C was used as an indicator of thermal tolerance, simulating febrile or intestinal conditions. Isolates that remained viable at 39 °C were considered suitable for further probiotic characterization.

#### 2.5.2. Acid Tolerance

To evaluate acid resistance, the isolates were exposed to modified MRS broth adjusted to pH 2.0 ± 0.2, 4.0 ± 0.2, and 6.0 ± 0.2 [20]. Bacterial cultures previously grown for 24 or 48 h at 37 °C were resuspended to an OD_600_ of 0.1 (1 × 10^8^ CFU/mL). In each well of a 96-well microplate, 100 μL of the pH-adjusted medium and 100 μL of the bacterial suspension were combined and incubated at 37 °C. Survival rates were calculated based on CFU/mL after 30 and 120 min of exposure.

#### 2.5.3. Bile Salt Tolerance

Bile salt resistance was assessed using MRS broth supplemented with 1% Oxgall [20]. The bacterial inoculum, prepared as described above, was added to the bile salt-containing medium in a 96-well plate and incubated at 37 °C. Viability was determined by comparing CFU/mL at 30 and 120 min to the initial count at time zero.

#### 2.5.4. Lysozyme Tolerance

To simulate exposure to lysozyme, the isolates were grown in 10 mL of MRS broth at 37 °C and centrifuged at 10,000× *g* for 10 min at room temperature (NT 825, Novatecnica, Rio de Janeiro, Brazil). The bacterial cells were washed twice and resuspended in 2 mL of phosphate-buffered saline (PBS). Bacterial suspension from each isolate (10^8^–10^9^ CFU/mL) was prepared into a sterile electrolyte solution (0.22 g/L CaCl_2_, 6.2 g/L NaCl, 2.2 g/L KCl, 1.2 g/L NaHCO_3_) containing 100 µg/mL lysozyme (Sigma-Aldrich, St. Louis, MO, USA) [23]. Survival was assessed by CFU/mL after 30 and 120 min, relative to the initial count.

### 2.6. Evaluation of Antimicrobial Activity of Lactiplantibacillus plantarum Isolates

The antimicrobial potential of the isolates was assessed using two complementary approaches: (i) the overlay assay and (ii) the well diffusion assay with cell-free supernatant collected after 24 h and 48 h of incubation. The tested pathogens were *Enterococcus faecalis* ATCC 29212, *Escherichia coli* 042, *Pseudomonas aeruginosa* ATCC 27856, *Salmonella enterica* ATCC 13076, and *Staphylococcus aureus* ATCC 6538.

To evaluate direct antagonism, each *L. plantarum* isolate was cultured in liquid MRS medium at 37 °C for 16 h. Cells were harvested by centrifugation (10,000× *g* for 10 min), washed twice with sterile PBS, and resuspended in PBS to a final concentration of 1 × 10^6^ CFU/mL. A 5 μL aliquot of this standardized suspension was spotted onto MRS agar plates and incubated at 37 °C for 24 h to allow colony development. After this period, a 2 mm layer of soft agar containing the respective pathogen (1 × 10^8^ CFU/mL) was overlaid on the plates, which were then incubated for an additional 24 h at 37 °C. Antimicrobial activity was assessed by measuring the diameter of inhibition zones surrounding the BAL colonies.

The inhibitory effects of metabolites secreted by the isolates were assessed using cell-free supernatants obtained from cultures after 24 h and 48 h of incubation. Each sample was centrifuged (10,000× *g* for 10 min) and passed through a 0.22 µm filter. Two conditions were tested: supernatant at its original pH (pH 4.0 ± 0.2) and supernatant adjusted to neutral pH (pH 7.0 ± 0.2). Mueller–Hinton agar plates were inoculated with the target pathogens, and wells were created using a sterile punch. Each well was filled with 10 μL of the respective supernatant. Plates were incubated at 37 °C for 24 h, and inhibition zones were measured. This assay allowed differentiation between pH-dependent and pH-independent inhibitory effects under both incubation times.

### 2.7. Multivariate Statistical Analysis

To assess the overall probiotic capacity and discriminate isolates based on simultaneous performance in in vitro tests, a Principal component analysis (PCA) was performed. Logarithmic variations (Δlog CFU) between the initial (0 min) and final (180 min) time points for bile salt and lysozyme tolerance assays, and between pH 6.8 and pH 2.7 for acid tolerance, were converted into retention fractions (10^Δlog^). Together with the mean antagonism halo diameters (mm), these values were standardized using Z-scores. The analysis was conducted in Python (v. 3.9) using the Scikit-learn library for algorithmic processing and Matplotlib (v. 3.10.7) for graphical generation of biplots (variable loadings and isolate scores). Explained variance and loadings were reported to interpret the contribution of each variable.

### 2.8. Genome Sequencing and Taxonomic Confirmation

The bacterial sample was shipped in NeoSample X buffer (Neoprospecta Microbiome Technologies, Florianópolis, Brazil). Genomic DNA was extracted using a proprietary magnetic bead-based protocol (Neoprospecta). Libraries were prepared with the Illumina DNA Prep—Nextera kit (Illumina, San Diego, CA, USA) and quantified using the Collibri Library Quantification Kit (Invitrogen, Carlsbad, CA, USA). Sequencing was performed on an Illumina NextSeq platform, generating paired-end reads (2 × 150 bp). Raw reads underwent quality filtering based on Phred scores, discarding sequences with Q < 20 and trimming adapters or low-quality regions. Post-QC assembly was conducted using the proprietary pipeline oneshotWGS v1.9, which integrates widely used bioinformatics tools. The workflow includes A5 assembly for adapter removal, error correction, and scaffold generation, followed by chimera removal to produce the best assembly. Assembly metrics (e.g., scaffold count, GC content, N50, L50) were calculated using QUAST v5.2.0, and genome annotation was performed with Prokka v1.14.6, supported by curated proprietary databases derived from Pfam, GenBank, and nt/nr. The assembly was classified as a draft genome, as expected for short-read sequencing, and metrics such as contig count, N50, and GC content were calculated using QUAST.

Taxonomic validation was performed in silico using the OrthoANIu algorithm (https://www.ezbiocloud.net/tools/ani) accessed on 3 November 2025. [24], which applies Average Nucleotide Identity (ANI) analysis to the best assembly against reference genomes. A cutoff of 97% ANI was adopted, consistent with the standard threshold for prokaryotic species delineation.

### 2.9. Multilocus Sequence Analysis

In addition to the ANI confirmation, the taxonomic identity of the isolate M2 was confirmed by multilocus phylogenetic analysis. The sequences of five phylogenetic markers were retrieved from the assembled genome for comparative analysis: 16S ribosomal RNA (16S rRNA), 23S ribosomal RNA (23S rRNA), DNA gyrase subunit B (*gyrB*), DNA gyrase/topoisomerase IV subunit A (*gyrA*), and RecA bacterial DNA recombination protein (*recA*). These sequences were aligned against reference strains of *L. plantarum* using BLASTn (https://blast.ncbi.nlm.nih.gov/Blast.cgi, accessed on 3 November 2025). Homology percentages were calculated to evaluate the discriminatory power of each marker. Based on the comparative results, the *gyrB* gene was selected for phylogenetic tree construction due to high resolution and consistent identity across multiple reference strains. Phylogenetic analysis was performed using MEGA X software (Molecular Evolutionary Genetics Analysis, version 12), with sequences aligned via ClustalW. The tree was constructed using the Neighbor-Joining method with 1000 bootstrap replicates. *E. faecalis* JCM 5803 and *E. coli* E1542 were used as outgroups.

### 2.10. Functional Annotation of the Genome

Functional annotation of the genome was performed using eggNOG-mapper v2.1.12 (DIAMOND mode) with the following parameters: *e*-value threshold of 1 × 10^−3^, minimum identity of 40%, and query/subject coverage ≥20%. The analysis retrieved orthology assignments and functional predictions across multiple databases, including COG, GO, KEGG, and Pfam. Annotations were exported in Excel format for downstream interpretation. Genes were classified according to COG functional categories and mapped to metabolic pathways, stress response systems, and cell surface structures relevant to probiotic functionality.

### 2.11. In Silico Analysis of Antibiotic Resistance and Virulence Genes

The chromosomal and plasmidial sequences were screened for acquired antibiotic resistance genes using ABRicate v. 1.0.1 against the CARD database (update 3 November 2025) with minimum identity and coverage thresholds of 90% and 80%, respectively. Virulence factors were assessed using ABRicate against the VFDB database (update 3 November 2025) under the same thresholds. Additionally, complementary protein-based analysis was performed using the CARD RGI web server (https://card.mcmaster.ca/analyze/blast, accessed on 3 November 2025) with the “protein homolog model” to identify distant homologs potentially associated with intrinsic resistance [25]. Hits were interpreted according to EFSA guidelines, considering only high-identity matches as evidence of acquired resistance.

### 2.12. Assessment of Phenotypic Traits Relevant to Probiotic Functionality of Lactiplantibacillus plantarum M2

#### 2.12.1. Hemolytic Activity

For hemolytic activity, the isolate M2 was cultured on blood agar plates containing 5% sheep blood and incubated at 37 °C for 48 h. Hemolysis was classified according to the appearance of lysis zones around colonies: (i) β-hemolysis corresponds to complete lysis of red blood cells, producing a clear and transparent zone; (ii) α-hemolysis indicates partial lysis, resulting in a greenish halo due to oxidation of hemoglobin to methemoglobin; and (iii) γ-hemolysis represents the absence of hemolysis, with no visible change in the medium [26].

#### 2.12.2. Biofilm Formation

Biofilm formation was evaluated in 96-well microplates using crystal violet staining. Isolate M2 was cultured in MRS broth at 37 °C for 48 h, then diluted in saline solution (0.9%) to a final concentration of 10^8^ CFU/mL. Each well received 180 μL of MRS broth and 20 μL of the bacterial suspension. Negative controls contained only 200 μL of MRS broth. Plates were incubated at 37 °C for 48 h. After incubation, wells were washed twice with PBS to remove non-adherent cells, fixed with 200 μL of methanol for 15 min, stained with 200 μL of crystal violet for 15 min, and rinsed with PBS. The dye was solubilized with 250 μL of ethanol, and 200 μL of the solution was transferred for absorbance reading at 630 nm (Plate reader MB-580; Heales, Shenzhen, China) [27]. Biofilm production was classified based on the optical density of the test (ODₜ) relative to the negative control (ODc), calculated as

$$\mathrm{Biofilm}\;\mathrm{classification}:\;\begin{array}{c} Negative\;(N):\;{OD}_{t}\leq ODc\\ Weak\;(W):\;{OD}_{c}<{OD}_{t}\leq 2\times ODc\\ Moderate\;(M):\;2\times ODc<{OD}_{t}\leq 4\times ODc\\ Strong\;(S):\;{OD}_{t}>4\times ODc \end{array}$$

#### 2.12.3. Autoaggregation Assay

Autoaggregation was evaluated by suspending *L. plantarum* M2 in PBS to an OD_600_ of 0.3. The suspension was incubated at 37 °C without agitation for 1 h. After incubation, the upper phase was collected, and its absorbance was measured at 600 nm [28]. Autoaggregation (%) was calculated as:

$$\mathrm{Autoaggregation}\;(\%)=\frac{\mathrm{Initial}\;\mathrm{OD}-\mathrm{Final}\;\mathrm{OD}}{\mathrm{Initial}\;\mathrm{OD}}\times 100$$

#### 2.12.4. Coaggregation Assay

Coaggregation with enteric pathogens (*E. coli* 042, *E. faecalis* ATCC 29212, and *S. enterica* ATCC 13076) was evaluated by mixing equal volumes (2 mL) of bacterial suspensions of the LAB isolate and each pathogen, both adjusted to OD_600_ = 0.3. Mixtures were incubated at 37 °C for 1 h without agitation [29]. Absorbance was measured at 600 nm, and coaggregation (%) was calculated using:

$$\mathrm{Coaggregation}\;(\%)=\frac{\left(\frac{A_{x}+A_{y}}{2}-A_{\left(x+y\right)}\right)}{\left(\frac{A_{x}+A_{y}}{2}\right)}\times 100$$

where $A_{x}$ = absorbance of the LAB isolate, $A_{y}$ = absorbance of the pathogen, and $A_{\left(x+y\right)}$= absorbance of the mixture.

### 2.13. Physicochemical Properties of Cell Surface of Lactiplantibacillus plantarum M2

Cell surface properties were determined using the Bacterial Adhesion to Solvent (BATS) method. Bacterial cells were harvested, washed, and resuspended in PBS (pH 7.2) to 10^8^ CFU/mL. A 3 mL aliquot of the suspension was mixed with 1 mL of solvent: toluene (nonpolar), chloroform (acidic polar), or ethyl acetate (basic polar). Mixtures were vortexed for 1 min and allowed to stand for 5 min for phase separation. The aqueous phase absorbance was measured at 600 nm [28]. Adhesion (%) was calculated as:

$$\mathrm{Adhesion}\;(\%)=(1-A/A_{0})\times 100$$

where $A_{0}$ = absorbance before solvent addition and A = absorbance after phase separation. Adhesion to toluene indicates hydrophobicity, while affinity to chloroform and ethyl acetate reflects electron donor/acceptor properties of the cell surface.

### 2.14. In Silico Proteome Hydropathy Analysis

The predicted proteome of *L. plantarum* M2 (N = 3226 proteins) was analyzed to assess the hydropathy profile of the cell surface using a custom Python script (based on Biopython, Pandas, and SciPy). Each protein was classified based on sequence features to determine its probable localization. Surface-contributing proteins (N = 199) were defined as those containing: (i) a predicted signal peptide (indicative of secretion, using a heuristic based on an N-terminal hydrophobic region followed by an A-X-A cleavage site); (ii) A lipoprotein motif (L(A/S)(A/G)C) near the signal peptide cleavage site; or (iii) A sortase-anchoring motif (LPXTG) near a C-terminal transmembrane domain and a positively charged tail. The Grand Average of Hydropathy (GRAVY) index was calculated for each protein using the Kyte-Doolittle scale. For secreted proteins, GRAVY was also calculated for the mature portion (post-cleavage). GRAVY distributions for the total proteome, the surface subset, and the non-surface (cytosolic) background were compared. Statistical significance of the differences was assessed using Welch’s *t*-test and the Mann–Whitney U test. Protein annotations within the surface subset were manually inspected to identify relevant functional domains (e.g., WxL, LysM, MucBP).

### 2.15. CAZyme Annotation

*The* predicted proteome of *L. plantarum* M2 (N = 3226 proteins) was submitted to the dbCAN3 web server (version 11.0; https://bcb.unl.edu/dbCAN2/index.php, accessed on 3 November 2025) for automated annotation of Carbohydrate-Active enZymes (CAZymes) [30]. Hits were identified and classified into CAZyme families (e.g., GH, GT, PL, CE, AA) based on the HMMER tool against the dbCAN HMM database.

### 2.16. Prediction of Antimicrobial Peptides and Secondary Metabolite Biosynthetic Gene Clusters (BCG)

The *L. plantarum* M2 genome was analyzed for antimicrobial BGCs using the BAGEL4 web server (http://bagel4.molgenrug.nl/, accessed on 3 November 2025) [31]. The analysis was performed on the full genome sequence to identify putative bacteriocin gene clusters and classify them based on the BAGEL4 database. For novel AMP prediction, the genome of *Lactiplantibacillus plantarum* M2 was analyzed using Macrel (version 1.5.0) [32] in a Conda environment (Bioconda channel) using the command ‘macrel contigs --fasta M2_Genome.fna --output macrel_out.’ Additionally, secondary metabolite BGCs were predicted locally with *antiSMASH* v7.1.0 under relaxed strictness [33]. Gene prediction was performed by *antiSMASH* using the Prodigal tool (--genefinding-tool prodigal). In silico minimum inhibitory concentrations (MICs) were predicted with APEX v.2 [34], an ensemble deep-learning learning model. The peptides were ranked by median MIC values against the 34 bacterial strains (Gram-negative, Gram-positive, and gut commensals).

### 2.17. Evaluation of the In Vivo Antimicrobial Effects of Lactiplantibacillus plantarum M2 Against Enteropathogens Using Tenebrio molitor Larvae

To investigate the in vivo antimicrobial efficacy of *L. plantarum* M2, an infection model using *Tenebrio molitor* larvae was employed. This model simulates host–pathogen interactions and allows assessment of probiotic protective effects under physiological conditions. The pathogens selected for in vivo testing were *E. coli* 042 and *S. enterica* ATCC 13076. These strains were chosen based on previous evidence indicating that *T. molitor* larvae are particularly susceptible to enteric Gram-negative bacteria, which cause measurable mortality in this model. Their high virulence in larvae makes them suitable for evaluating the protective effects of probiotic candidates.

Larvae were obtained commercially from Biofábrica (São Luís, Brazil) and maintained on a cornmeal-based diet provided by the supplier until the start of the acclimatization period. Two experimental approaches were used: prophylactic treatment (administration of M2 before infection) and post-infection treatment (administration of M2 after pathogen challenge). Early-stage *T. molitor* larvae (~100 mg), selected for uniform size and absence of color alterations, were used in all assays. Individuals exhibiting dark spots or signs of melanization were excluded. Before infection, larvae were acclimatized for 2 h in sterile Petri dishes at 37 °C, protected from light, without food. Larvae were infected with 10 μL of a bacterial suspension (OD_600_ = 0.1; ~1.0 × 10^8^ CFU/mL) injected into the intersegmental membrane between the second and third abdominal segments. In the prophylactic group, *L. plantarum* M2 was administered 2 h before infection; in the post-infection group, it was administered 2 h after pathogen inoculation. A PBS-injected group served as the viability control. Larval survival was monitored daily for 10 days, and mortality was defined by the absence of movement in response to stimulation. This assay enabled the evaluation of the protective potential of isolate M2 against enteric infections, as well as its safety profile in a live host model.

### 2.18. Statistical Analysis

All results were expressed as mean ± standard deviation (SD). The assays were performed in quadruplicate with two independent repetitions. Inhibition percentages and other quantitative outcomes were calculated as the average values obtained from independent experiments. Statistical comparisons were performed using GraphPad Prism (version 10.0). Parametric data were analyzed using Student’s *t*-test, one-way ANOVA, or two-way ANOVA, followed by Bonferroni post hoc tests when applicable. For survival analysis, Kaplan–Meier survival curves were generated and compared using the Log-Rank test. A significance level of *p* < 0.05 was adopted for all analyses.

## 3. Results

### 3.1. Isolation and Identification of Lactobacilli Isolates

Following the spontaneous fermentation of the fruit samples listed in Table 1, a total of 14 microbial isolates were obtained. The majority were identified as yeasts, while four isolates were Gram-positive bacilli. Preliminary screening was performed using Gram staining and catalase testing, followed by definitive identification through MALDI-TOF MS. Three isolates recovered from *H. balsamifera* were identified as *Lactiplantibacillus plantarum*, and they were designated M1, M2, and M4. The remaining isolates included various yeast species such as *Saccharomyces cerevisiae*, *Wickerhamomyces anomalus*, *Pichia manshurica*, *Yarrowia lipolytica*, and *Saccharomycodes ludwigii*. These results highlight *H. balsamifera* as a promising source of LAB, particularly *L. plantarum*, which was not detected in the other fruit samples.

### 3.2. Antimicrobial Susceptibility Profile of Lactiplantibacillus plantarum Isolates

The antimicrobial susceptibility of isolates M1, M2, and M4 was evaluated using the disc diffusion method, and the results are summarized in Table 2. Overall, the isolates demonstrated a high sensitivity profile, with 75% of the tested antibiotics showing inhibitory activity. All three isolates were sensitive to ampicillin, ceftazidime, erythromycin, clindamycin, rifampicin, and penicillin. Resistance was observed only to gentamicin and vancomycin, which are known to exhibit limited efficacy against certain LAB due to intrinsic resistance mechanisms. These findings support the safety profile of the isolates, as sensitivity to clinically relevant antibiotics is a key criterion for probiotic candidacy, particularly to avoid horizontal gene transfer of resistance traits.

### 3.3. Lactiplantibacillus plantarum Isolates Exhibit High Resilience to All Tested Physiological Stressors

The ability of isolates M1, M2, and M4 to withstand physiologically relevant stress conditions was assessed through thermal, bile salt, lysozyme, and acid tolerance assays. All three isolates remained viable at 39 °C, indicating their capacity to grow under elevated temperatures that mimic febrile or intestinal conditions.

**Figure 1:**
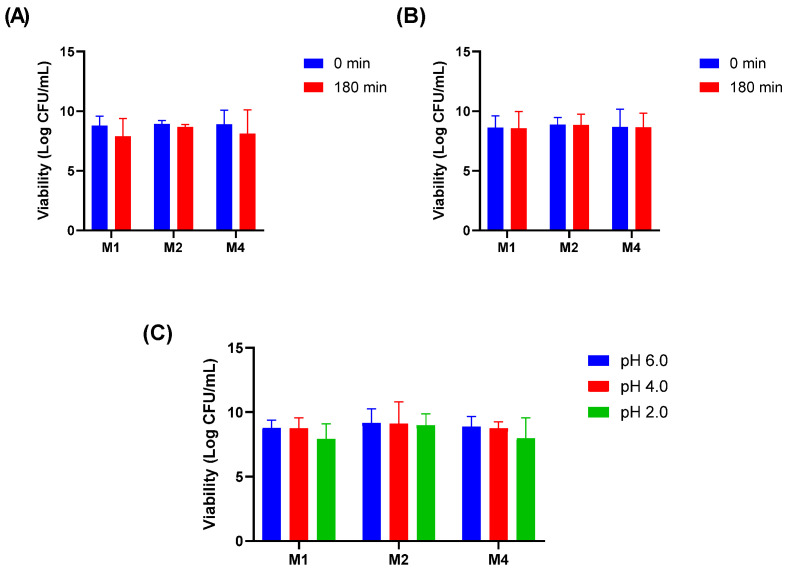
Tolerance of *Lactiplantibacillus plantarum* isolates to physiological stressors. (**A**) Bile salt tolerance (1% Oxgall); (**B**) Lysozyme resistance in sterile electrolyte solution; (**C**) Acid tolerance at pH 2.0, 4.0, and 6.0.

In the bile salt tolerance assay, the isolates maintained their viability after 180 min of exposure to 1% Oxgall, demonstrating their potential to survive passage through the small intestine (Figure 1A). Similarly, the isolates exhibited resistance to lysozyme when suspended in sterile electrolyte solution, remaining viable after 180 min of exposure (Figure 1B). Acid tolerance was evaluated at pH 2.0, 4.0, and 6.0. Although a slight reduction in CFU/mL was observed at pH 2.0, all isolates remained viable, suggesting their ability to survive the acidic conditions of the stomach (Figure 1C).

### 3.4. Lactiplantibacillus plantarum Isolates Have Antimicrobial Activity Against Selected Pathogens

**Figure 2:**
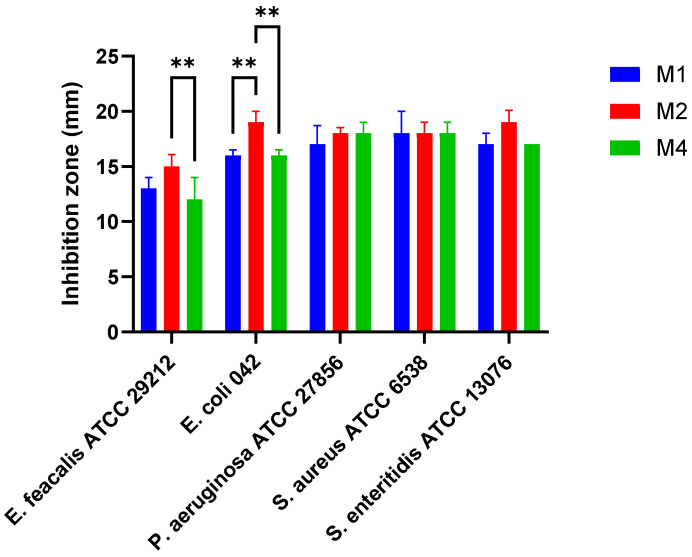
Antimicrobial activity of *Lactiplantibacillus plantarum* isolates towards pathogenic bacteria. ** *p* < 0.01.

The isolates M1, M2, and M4 exhibited antimicrobial activity against *E. faecalis* ATCC 29212 (inhibition zones ranged from 12 ± 2.0 to 15 ± 1.1 mm), *E. coli* 042 (16 ± 0.5 to 19 ± 1.0 mm), *P. aeruginosa* ATCC 27856 (17 ± 1.1 to 18 ± 1.0 mm), *S. aureus* ATCC 6538 (18 mm for all isolates), and *S. enteritidis* ATCC 13076 (17 ± 0.0 to 19 ± 1.1 mm) (Figure 2). Among the tested strains, *L. plantarum* M2 demonstrated the most pronounced antimicrobial activity, with significantly larger inhibition zones against *E. coli* 042 and *E. faecalis* ATCC 29212 (*p* < 0.01).

In addition to the overlay assay, the antimicrobial activity of the isolates was also evaluated using the well diffusion method with cell-free supernatants obtained after 24 h and 48 h incubation. Under the tested conditions—both at native acidic pH (4.0 ± 0.2) and after neutralization (pH 7.0 ± 0.2)—none of the isolates produced inhibition zones against the target pathogens. These results indicate that the antimicrobial effect observed in the overlay assay is likely dependent on direct cell–pathogen interactions, rather than the secretion of diffusible antimicrobial compounds such as organic acids or bacteriocins.

### 3.5. Comparative Analysis of Probiotic Profile (PCA)

**Figure 3:**
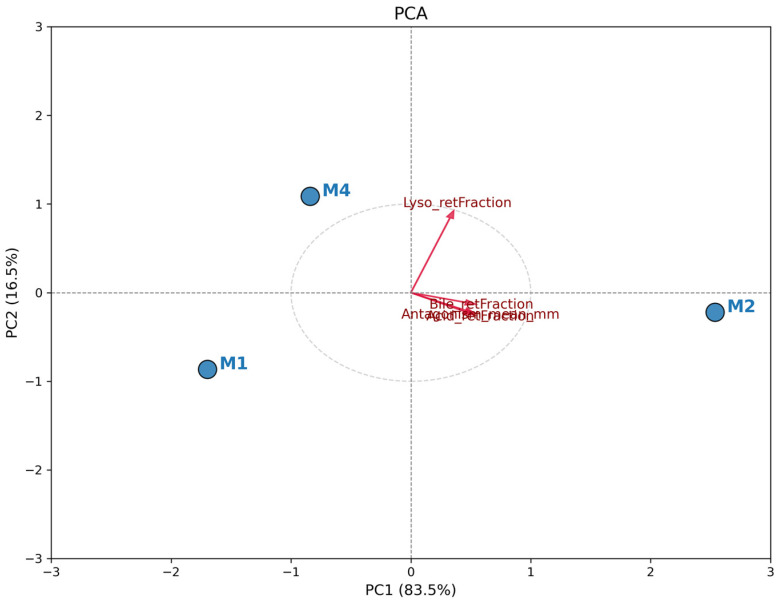
Principal Component Analysis (PCA) biplot illustrating the probiotic potential of *Lactiplantibacillus plantarum* isolates. The plot displays the spatial distribution of isolates M1, M2, and M4 (blue dots) relative to the loading vectors of the evaluated variables (red arrows). Variables include mean antagonism activity against pathogens (mm) and retention fractions (survival rates) under acidic conditions (pH 2.7), bile salts, and lysozyme stress.

The PCA revealed a clear distinction among *L. plantarum* isolates based on the evaluated functional characteristics. Figure 3 shows the biplot, where the total data variation was predominantly explained by the first principal component (PC1), indicating a strong unidirectional correlation between resistance attributes and antagonistic activity. Isolate M2 exhibited a distinct and superior probiotic profile, positioned positively along the PC1 axis (right quadrant). The vector projection of all analyzed variables (Antagonism, Acid, Bile, and Lysozyme Resistance) strongly points toward isolate M2. This indicates that this isolate simultaneously combined the largest inhibition halos and the highest survival rates under simulated gastrointestinal stress conditions. In contrast, isolates M1 and M4 clustered on the opposite side of the graph (negative PC1), showing a similar functional profile to each other but inferior to M2 under the tested conditions. The spatial proximity between M1 and M4 suggests that these isolates share less robust resistance and antimicrobial activity phenotypes compared to the isolate obtained from spontaneous fermentation (*L. plantarum* M2). Therefore, PCA confirms that M2 is the most promising candidate for biotechnological application among the evaluated strains and it was selected for the further assays.

### 3.6. Lactiplantibacillus plantarum M2 Has Traits Relevant to Probiotic Functionality 

**Figure 4:**
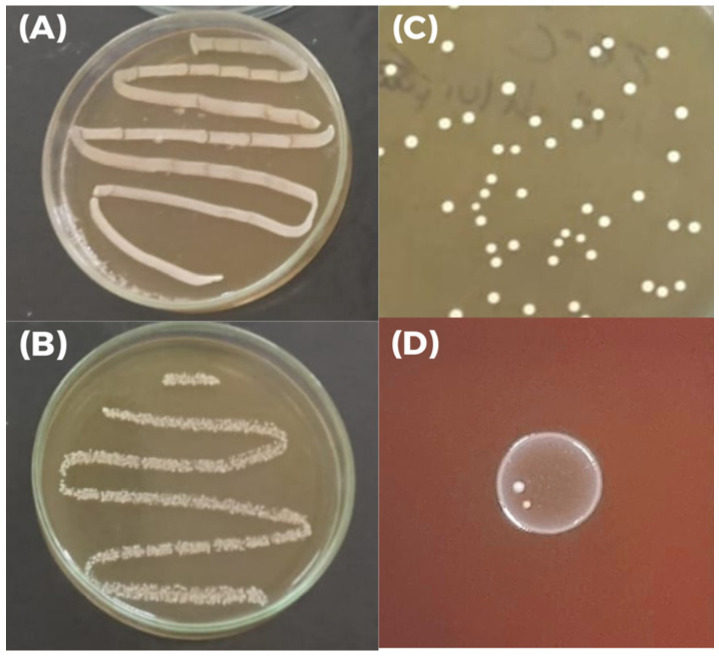
Morphological and growth characteristics of *Lactiplantibacillus plantarum* M2 under different conditions. (**A**) Growth of *L. plantarum* M2 under aerobic conditions on MRS agar; (**B**) Growth of *L. plantarum* M2 under anaerobic conditions on MRS agar; (**C**) Colonies of *L. plantarum* M2 MRS agar plates showing typical morphology: round, opaque, and milky white; (**D**) Hemolytic activity of *L. plantarum* M2 on blood agar indicating γ-hemolysis (absence of lysis zones).

Growth of *L. plantarum* M2 was observed under both aerobic and anaerobic conditions, confirming its facultative anaerobic nature (Figure 4A,B). Its colonies on MRS agar plates are round, opaque, and milky white, exhibiting typical characteristics of LAB (Figure 4C). Additionally, the isolate showed γ-hemolysis on blood agar, indicating an absence of hemolytic activity and supporting its safety profile (Figure 4D). In the biofilm formation assay, the isolate M2 exhibited an optical density 2.74-fold higher than the negative control (ODc = 0.08025 ± 0.001, and ODt = 0.21975 ± 0.097; *p* < 0.05), classifying it as a moderate biofilm producer according to the established criteria.

### 3.7. Genomic Assembly, Taxonomic Confirmation, and In Silico Safety Assessment of Lactiplantibacillus plantarum M2

The genomic sequence assembly of strain M2 resulted in a draft genome comprising 64 contigs, with a total size of 3,403,182 bp, GC content = 44.245%, N50 = 119,828 bp (L50 = 9) and N90 = 35,411 bp (L90 = 30); the proportion of ambiguous bases was 0.014%. These values are consistent with *L. plantarum* genomes reported in the literature (~3.25–3.50 Mb; ~44% GC), including the reference strain WCFS1 (~3.40 Mb; ~44.47% GC) and the type strain DSM 20174 (~3.24 Mb; ~44.52% GC). Species confirmation was based on ANI, adopting the ≥97% threshold recommended for interspecific delineation. The ANI calculated using OrthoANIu was 99.14% when comparing *L. plantarum* M2 to *L. plantarum* DSM 20174 and 99.13% against the *L. plantarum* WCFS1, with reciprocal alignment coverage of ~65–68%, which strongly supports the classification of M2 as *L. plantarum*. This Whole Genome Shotgun project has been deposited at DDBJ/ENA/GenBank under the accession number JBQOUW000000000, BioProject PRJNA1303982, and BioSample SAMN50545230. In addition to the chromosomal assembly, genome analysis revealed the presence of one plasmid (designated plasmid_AB384) with a size of 19,586 bp, GC content of 39.362%, and a single contig (N50 = 19,586 bp; L50 = 1). The proportion of ambiguous bases was 0.005%.

Screening with ABRicate against the CARD database (BLASTn; thresholds ≥ 90% identity and ≥80% coverage) detected no acquired antibiotic resistance genes in the chromosomal assembly or in plasmid. Complementary analysis using the CARD RGI webserver identified a distant homolog of *vanY* (vanB cluster) with ~32% amino acid identity and ~96% length coverage, classified as *strict*. This pattern is consistent with intrinsic glycopeptide tolerance described for lactic acid bacteria and not plasmid-borne, aligning with the vancomycin-resistant phenotype while indicating no evidence of acquired, transferable determinants. Screening against the VFDB database revealed no virulence genes in either the chromosomal or plasmid sequences, supporting the safety profile of strain M2.

### 3.8. Multilocus Phylogenetic Analysis of Lactiplantibacillus plantarum M2

The isolate M2 exhibited high sequence homology with *Lactiplantibacillus* reference strains across all five markers (Table 3). The 16S rRNA and 23S rRNA genes showed homology values ranging from 99.32% to 99.66%, confirming genus-level classification. The *recA* and *parC* genes also demonstrated high identity, supporting species-level identification. Among the markers, the *gyrB* gene presented the most consistent and highest homology (≥99.5%) across multiple strains, including NBRC15891T and JCM1149T. Due to its superior phylogenetic resolution, *gyrB* was selected for subsequent tree-based analysis to support the classification of isolate M2.

**Figure 5:**
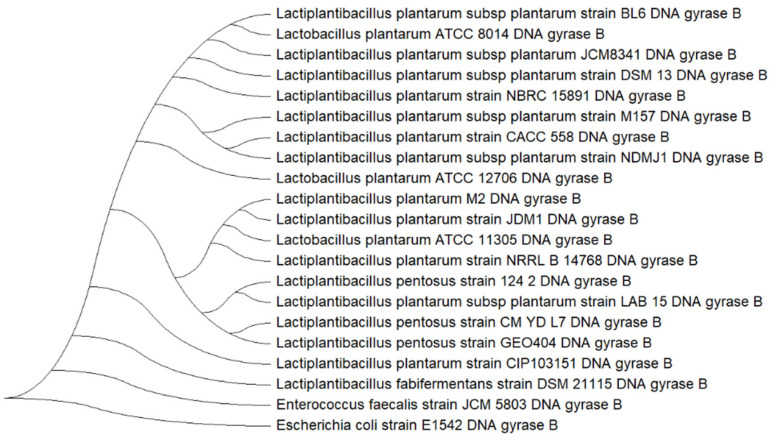
Phylogenetic tree based on the DNA gyrase B (*gyrB*) gene sequences showing the relationship between isolate M2 and reference strains of *Lactiplantibacillus plantarum*. The tree was constructed using the Neighbor-Joining method with 1000 bootstrap replicates in MEGA X. *Enterococcus faecalis* JCM 5803 and *Escherichia coli* E1542 were used as outgroups.

The phylogenetic tree constructed using *gyrB* gene (Figure 5) revealed a clear clustering of isolate M2 within the *L. plantarum* clade. Isolate M2 grouped closely with reference strains such as *L. plantarum* NBRC 15891, DSM 13, and JCM 8341, indicating high genetic similarity. The tree also distinguished *L. plantarum* from closely related species such as *Lactiplantibacillus pentosus* and *Lactiplantibacillus fabifermentans*, which formed separate branches. The inclusion of *E. faecalis* JCM 5803 and *E. coli* E1542 as an outgroup provided additional support for the phylogenetic placement of M2 within the *L. plantarum* lineage.

### 3.9. Functional Profiling of Lactiplantibacillus plantarum M2

The draft annotation revealed a broad functional repertoire consistent with adaptation to host and environmental niches. Multiple genes encoding cell surface adhesion domains were identified, including WxL, LysM, and MucBP, as well as LPXTG/CnaB anchoring motifs, suggesting strong potential for mucosal adhesion and biofilm formation. Genes involved in exopolysaccharide and teichoic acid biosynthesis (*tagE*, *rfbX*, *ica1*, *gtcA1*) and export systems (*tagG*/*tagH*) were detected, supporting the structural complexity of the cell envelope. Stress tolerance was evidenced by operons for osmoprotection (Opu/Cho systems), mechanosensitive channels (*mscL*), and metal ion transport (*feoAB*, *mntH*), alongside Fe-S cluster assembly proteins (*sufABCD*, *iscU*). A robust DNA repair toolkit was present, including *recN*, *recG*, *fpg*, *polA*, and *sbcCD*, which may contribute to survival under oxidative and acid stress. Regulatory elements for bacteriocin production (*pltR*, *pltK*) and immunity proteases (CAAX family) were also annotated, consistent with previously predicted plantaricin clusters. Additionally, complete pathways for riboflavin biosynthesis (*ribA*, *ribD*, *ribE*, *ribH*) were identified, indicating potential for vitamin B2 production.

### 3.10. Surface Properties and Aggregation Behavior of Lactiplantibacillus plantarum M2

The aggregation capacity and physicochemical surface properties of *L. plantarum* M2 were evaluated through autoaggregation, coaggregation with enteropathogenic bacteria, and the BATS assay (Table 4). The isolate exhibited low autoaggregation (7.8 ± 2.1%), suggesting limited ability to form homogeneous aggregates. Conversely, coaggregation values were markedly higher for *S. enterica* (21.1 ± 7.3%) and *E. faecalis* (20.2 ± 0.4%), indicating strong heterotypic interactions with these pathogens, which are relevant for competitive exclusion. The affinity profile obtained by BATS revealed negative adhesion to toluene (−4.37% to −24.41%), confirming a predominantly hydrophilic surface, while adhesion to chloroform and ethyl acetate remained below 4%, suggesting weak electron donor and acceptor properties. These findings imply that the antagonistic potential of M2 relies more on specific molecular interactions (e.g., surface proteins, exopolysaccharides) than on hydrophobic forces.

### 3.11. In Silico Validation of Hydrophilicity in Lactiplantibacillus plantarum M2

**Figure 6:**
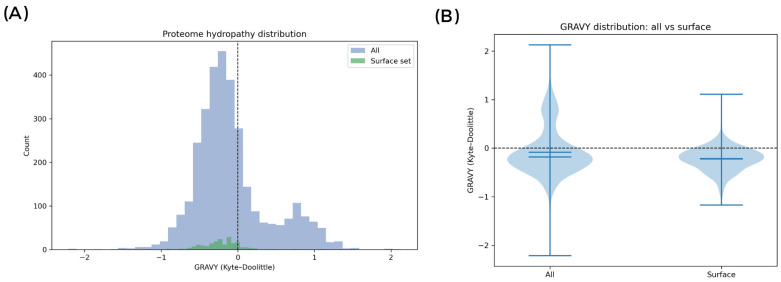
In silico hydropathy analysis of the *Lactiplantibacillus plantarum* M2 proteome. (**A**) Histogram of GRAVY index (Kyte–Doolittle scale) for all predicted proteins (blue) versus the surface-contributing subset (green). (**B**) Violin plot comparing GRAVY distributions between the whole proteome and the surface subset.

To independently validate whether the molecular inventory exposed at the cell interface is biased toward hydrophilicity (Figure 6), the M2 proteome was profiled (3226 proteins) and contrasted with a subset expected to contribute to surface exposure (secreted, lipoproteins, LPXTG-anchored; N = 199). The surface subset exhibited a left-shifted GRAVY distribution (median −0.2148; IQR −0.3519 to −0.0792) relative to the whole proteome (median −0.1828; IQR −0.3840 to 0.0721), with a significantly more negative mean hydropathy (Welch’s *t*-test *p* = 1.4 × 10^−10^; Mann–Whitney *p* = 0.0123). The effect remained significant when the surface subset was compared against the non-surface background (Welch’s *t*-test *p* = 1.41 × 10^−11^; Mann–Whitney *p* = 0.0078). Notably, 84.9% of the surface proteins had GRAVY < 0 versus 69.4% in the proteome background.

Among surface-contributing proteins, two LPXTG sortase-anchored candidates were identified (BioY family protein and ECF-type riboflavin transporter, S component), and a single lipoprotein (FMN-binding domain protein). Among secreted proteins (N = 196), the median GRAVY further decreased after signal-peptide cleavage (−0.2466 vs. −0.2171), indicating that the matured exported moiety is, on average, more hydrophilic. Several showed extreme hydrophilicities after signal peptide removal (GRAVY_after_sp ≤ −0.5), suggesting strong solubility and potential adhesive roles. The most hydrophilic proteins were: hypothetical protein (GRAVY_after_sp = −1.361), HB1/ASXL restriction endonuclease HTH domain protein (−1.245), MobA/MobL family protein (−0.987), Biofilm formation stimulator VEG (−0.752), and Ribosomal prokaryotic L21 protein (−0.747).

Additionally, proteins with cell-wall binding domains were identified, including WxL domain proteins, LysM domain proteins, and MucBP domain proteins), which may mediate adhesion and aggregation through non-covalent interactions. Altogether, the convergence between BATS and in silico data supports a model in which M2 contact-dependent antagonism relies predominantly on specific surface macromolecules (e.g., cell-wall-anchored adhesins and/or exopolysaccharides) rather than nonspecific-hydrophobic forces.

### 3.12. Lactiplantibacillus plantarum M2 Possesses a Rich Genomic Repertoire for Polysaccharide Biosynthesis

**Figure 7:**
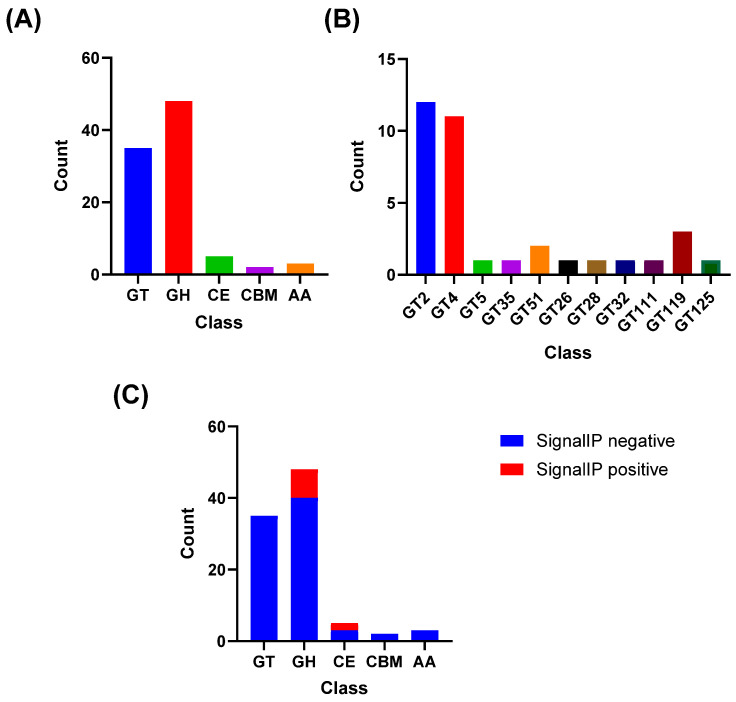
CAZyme repertoire and secretion signals in *Lactiplantibacillus plantarum* M2. (**A**) Distribution of CAZymes by class (GT, GH, CE, CBM, AA). (**B**) Counts of glycosyltransferase families associated with EPS biosynthesis (GT2, GT4, GT5, GT26, GT28, GT32, GT35, GT51, GT111, GT119, GT125). (**C**) CAZymes with and without predicted signal peptides (SignalP) by class, shown as stacked columns.

To confirm the hypothesis that surface polysaccharides are key components of the M2 cell envelope, the proteome was analyzed for CAZymes. The analysis identified that the CAZyme repertoire of *L. plantarum* M2 was dominated by glycosyl hydrolases (GH) and glycosyltransferases (GT), with 48 GH and 35 GT, while carbohydrate esterases (CE), auxiliary activity enzymes (AA), and carbohydrate-binding modules (CBM) were less represented (5, 3, and 2, respectively; Figure 7A). Within GTs, families associated with exopolysaccharides (EPS) biosynthesis were the most frequent, notably GT2 (n = 12) and GT4 (n = 11), followed by sporadic occurrences of GT119 (n = 3), GT51 (n = 2), and single representatives of GT5, GT26, GT28, GT32, GT35, GT111, and GT125 (Figure 7B). This profile supports the potential of *L. plantarum* to assemble EPS through classical glycosyl elongation and polymerization pathways.

Signal peptide prediction (SignalP) indicated that 10 CAZymes carry secretion signals, distributed mainly among GH (n = 8) and CE (n = 2); GT, AA, and CBM showed no signal peptides (Figure 7C). This pattern suggests that secreted enzymes involved in extracellular carbohydrate remodeling are predominantly hydrolases and esterases in the analyzed set.

### 3.13. Lactiplantibacillus plantarum M2 Genome Harbors Two Distinct Plantaricin Gene Clusters

**Figure 8:**
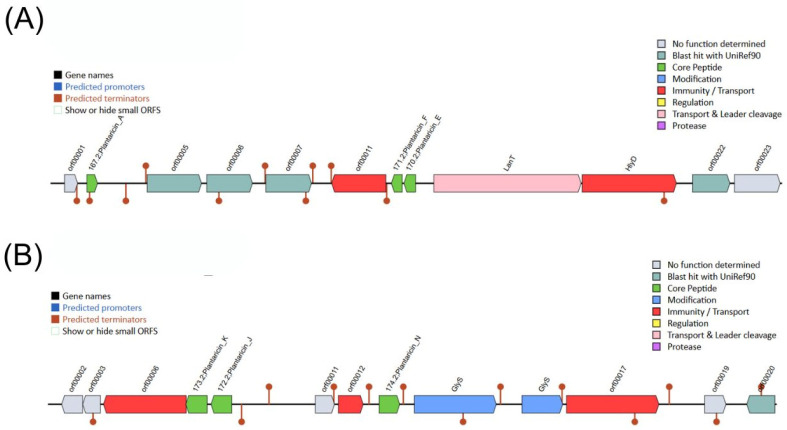
Genetic organization of plantaricin biosynthetic gene clusters (BGCs) in *L. plantarum* M2. (**A**) BGC 1 contains genes for PlnA (PF08129; PF10439), PlnF (PF04369; PF10439), PlnE (no Pfam hit shown in the image), a histidine kinase and response regulators PlnC/PlnD (PF00072), immunity PlnI (CAAX protease), ABC exporter LanT/PlnG (PF00005; PF03412) and accessory factor HlyD/PlnH (PF13437). (**B**) BGC 2 includes genes for PlnK (PF03047; PF10439), PlnJ (PF10439), PlnN (PF10439), immunity PlnM and PlnI, and the biosynthesis/processing protein PlnO (TIGR04195; PF00535).

To investigate the molecular basis for the strong contact-dependent antagonism observed in vitro, the M2 genome was mined for bacteriocin biosynthetic gene clusters (BGCs). The BAGEL4 analysis identified two distinct Class IIa bacteriocin BGCs. The first cluster encoded genes for Plantaricins A, E, and F (Pln A/E/F), while the second encoded Plantaricins J, K, and N (Pln J/K/N) (Figure 8). Both clusters included associated genes for ABC transporters and two-component regulatory systems (Histidine Kinase/Response Regulator), indicating complete and functional systems for bacteriocin export and regulation. Among the plantaricins, PlnA and PlnK showed the lowest predicted median MIC (90.49 µM and 94.77 µM), followed by PlnF (112.67 µM), PlnK (115.89 µM), PlnJ (119.31 µM) and PlnN (124.63 µM).

### 3.14. Lactiplantibacillus plantarum M2 Genome Encodes Two Antimicrobial Peptides

Two candidate antimicrobial peptides were identified in the genome of *L. plantarum* M2. The first peptide (23 aa) exhibited an AMP probability of 0.624 but a high hemolytic probability (0.901), suggesting limited therapeutic applicability without structural optimization. The second peptide (32 aa) showed an AMP probability of 0.505 and a lower hemolytic probability (0.386), indicating a safer profile. Both peptides belong to the CLP family, reinforcing the hypothesis that M2 harbors genes encoding antimicrobial peptides beyond canonical bacteriocin clusters detected by BAGEL4. BLASTp (https://blast.ncbi.nlm.nih.gov/Blast.cgi, accessed on 3 November 2025) analysis of the predicted AMPs revealed that the 32-aa peptide shares high identity (up to 100%) with hypothetical proteins from *Lactiplantibacillus* species, suggesting a conserved but uncharacterized function. In contrast, the 23-aa peptide aligned with diverse protein fragments from unrelated taxa, indicating that it may represent a non-specific sequence or require structural optimization to exhibit antimicrobial activity. Both AMPs presented predicted median MIC above 100 µM.

### 3.15. Lactiplantibacillus plantarum M2 Genome Harbors Four Unique Biosynthetic Clusters

Genome mining revealed four biosynthetic gene clusters (BGCs) distributed across distinct contigs (Table 5). The clusters were classified as RiPP-like, terpene, cyclic-lactone autoinducer, and type III polyketide synthase (T3PKS). The RiPP-like cluster (Region 9.1), located on contig 9, did not show significant similarity to any known BGCs in the MIBiG database. This cluster encodes a hypothetical precursor protein (67 aa) highly conserved among *Lactiplantibacillus* species (up to 97% identity), but with no functional annotation in public databases. Its genomic context (comprising CAAX proteases, ABC transporters, and secretion proteins) suggests a ribosomally synthesized peptide undergoing post-translational modifications, consistent with RiPP biosynthetic systems.

The terpene cluster (Region 10.1) may encode enzymes involved in terpenoid biosynthesis, which are often linked to membrane modulation and signaling. The cyclic-lactone autoinducer cluster (Region 12.1) is associated with quorum-sensing molecules, indicating possible roles in intercellular communication and regulation of competitive traits. Finally, the T3PKS cluster (Region 27.1) represents a polyketide biosynthetic locus, which could contribute to ecological fitness and antagonistic activity. None of the clusters exhibited high similarity to characterized entries in MIBiG, reinforcing the hypothesis that strain M2 harbors unique biosynthetic potential beyond canonical bacteriocins.

### 3.16. Lactiplantibacillus plantarum M2 Enhances Survival of Tenebrio molitor Larvae Challenged with Enteropathogenic Bacteria

**Figure 9:**
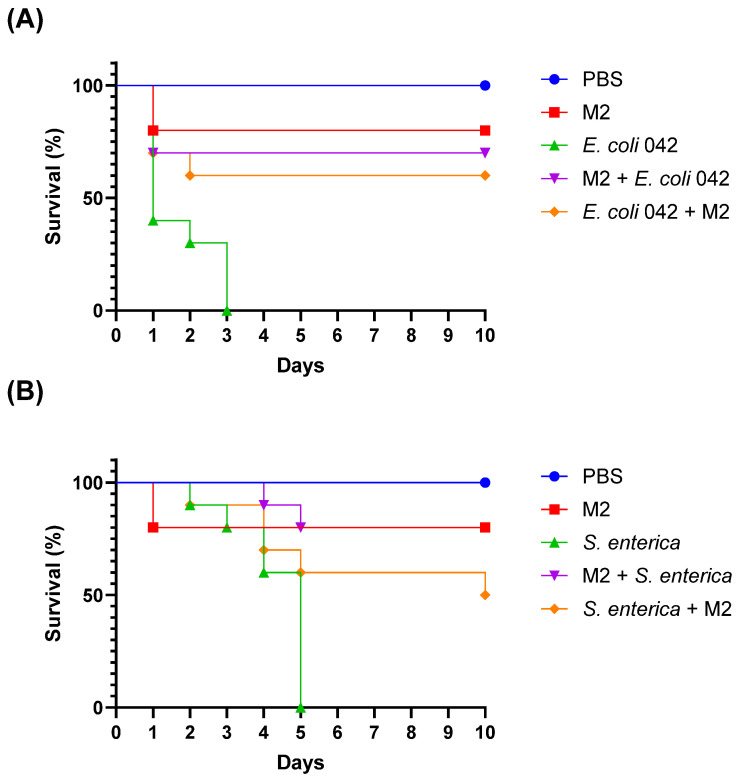
Survival curves of *Tenebrio molitor* larvae challenged with enteropathogenic bacteria. (**A**) Survival curves of larvae infected with *Escherichia coli* 042. Groups include PBS control, M2 alone, infection alone (*E. coli* 042), prophylactic treatment (M2 administered 2 h before infection), and post-infection treatment (M2 administered 2 h after infection). (**B**) Survival curves of larvae infected with *Salmonella enterica* ATCC 13076. Groups include PBS control, M2 alone, infection alone (*S. enterica*), prophylactic treatment, and post-infection treatment. Survival was monitored daily for 10 days.

To evaluate the protective effect of the isolate *L. plantarum* M2 against enteropathogens, *T. molitor* larvae were infected with *E. coli* 042 and *S. enterica* ATCC 13076, and treated either prophylactically or post-infection with M2. Survival was monitored daily for 10 days. In the *E. coli* 042 infection model, the control group (infected without treatment) exhibited 100% mortality within 24 h, with a median survival time of 1 day. In contrast, larvae treated with M2 showed significantly improved survival. The prophylactic group (M2 administered 2 h before infection) had a final survival rate of 70%, while the post-infection treatment group (M2 administered 2 h after infection) reached 60%. Both treated groups had undefined median survival times, indicating that more than 50% of the larvae survived until the end of the experiment (Figure 9A).

Larvae infected with *S. enterica* ATCC 13076 without treatment showed a median survival of 5 days and a final survival rate of 0%. The prophylactic treatment with M2 resulted in complete protection, with 80% survival and undefined median survival. The post-infection treatment group showed a median survival of 10 days and a final survival rate of 50%, demonstrating a partial protective effect (Figure 9B).

Importantly, the group treated only with M2 (without pathogen challenge) showed no mortality throughout the experiment, confirming that the isolate does not induce toxicity or adverse effects in the host model (Figure 5). These results indicate that isolate M2 exhibits significant in vivo antimicrobial activity, especially when administered prophylactically, and is capable of enhancing host survival against enteric bacterial infections without compromising host viability.

## 4. Discussion

The escalating demand for novel probiotic strains, particularly those suitable for advanced therapeutic delivery systems, underscores the importance of bioprospecting in tropical ecosystems such as those found in the Legal Amazon, which harbor diverse microbial communities and impose selective pressures that shape functional traits [7,8,13,35]. While most commercial strains are derived from well-characterized sources such as dairy or the human gut [13,14], limiting their ecological adaptability and functional diversity, plant-derived strains adapted to tropical niches may exhibit enhanced stress tolerance and adhesion capabilities—key attributes for gastrointestinal survival and pathogen exclusion [12,18]. Specifically, the isolation of *L. plantarum* M2 from this unique matrix is relevant, as its environmental origin suggests pre-adaptation to stress conditions that may translate into superior resilience during gastrointestinal transit and greater stability for pharmaceutical formulations. Therefore, this study focused on characterizing M2 to determine whether its functional profile offers distinct advantages over conventional probiotic sources.

*L. plantarum* is a LAB species extensively studied for its probiotic potential in food systems [36], with over three decades of research supporting its safety and efficacy [37]. Additionally, strains of *L. plantarum* exhibit antagonistic activity against a range of enteric pathogens, including *E. coli*, *Cronobacter sakazakii* [38], and *S. enterica* serovar Typhimurium [39], reinforcing their relevance as multifunctional probiotic candidates. Within this context, fruits of *H. balsamifera* (mirim) emerged as a particularly promising source of LAB. *H. balsamifera* belongs to the family Humiriaceae, widely distributed throughout the Amazon region [40]. It is characterized by its hard, aromatic wood, often exuding a balsamic resin, and by its drupaceous fruits with a fibrous mesocarp and a distinctive fragrance [41,42]. Traditionally, the plant has been used by local communities for its medicinal properties, which include scientifically validated anti-inflammatory, antioxidant, and antimicrobial effects [43,44,45]. The identification of three *L. plantarum* strains (M1, M2, and M4) from its fruits reinforces the relevance of *H. balsamifera* as a natural matrix for the bioprospecting of probiotic microorganisms with potential applications in human health, food safety, and the development of bio-based products. Among them, the isolate M2 was considered the most promising for probiotic application.

Taxonomic identity of isolate M2 was confirmed by ANI analysis, which yielded values above 99% against reference genomes of *L. plantarum*, exceeding the 97% threshold for species delineation. This high ANI score provides strong evidence for species-level classification and ensures traceability for comparative genomics and regulatory purposes. To complement this, a multilocus phylogenetic analysis using five markers (16S rRNA, 23S rRNA, *gyrA*, *gyrB*, and *recA*) was performed [18,46], with *gyrB* offering superior resolution and clustering M2 tightly within the *L. plantarum* clade. Together, these approaches validate the identity of M2 and support the interpretation of its functional genomic traits [1].

The antimicrobial susceptibility profile of the *L. plantarum* M2 (and other isolates) revealed high sensitivity to 75% of the antibiotics tested, including ampicillin, ceftazidime, erythromycin, clindamycin, rifampicin, and penicillin. Resistance was observed only to gentamicin and vancomycin, which is consistent with intrinsic resistance mechanisms commonly found in LAB and does not pose a significant risk of horizontal gene transfer [47], in accordance with EFSA safety guidelines for probiotics [48]. This phenomenon has been well-documented in various LAB strains isolated globally, including those from fermented foods and human sources [49,50]. Importantly, genomic analysis confirmed the absence of acquired resistance genes and virulence factors, reinforcing the safety of M2 for food and therapeutic applications. These findings support the safety of the isolates for use in food and therapeutic formulations.

Beyond safety, the ability to withstand physiological stressors is essential for probiotic functionality [51]. All *H. balsamifera*-derived strains remained viable at elevated temperatures (39 °C), mimicking febrile conditions, and resisted low pH (2.0) and the presence of bile salts and lysozyme, which are key barriers to survival during gastric transit and intestinal colonization [2]. This remarkable resilience is attributed to a series of sophisticated cellular adaptations. For instance, acid tolerance is maintained through the activation of proton pumps that actively expel hydrogen ions to keep intracellular pH near neutrality [52]. Meanwhile, bile salt tolerance is mediated by enzymes such as bile salt hydrolases (BSH), which detoxify these natural detergents that would otherwise damage cell membranes [53]. Furthermore, resistance to elevated temperatures is supported by the expression of heat shock proteins that protect cellular structures from denaturation [51].

These phenotypic traits are corroborated by genomic findings, since M2 harbors operons for osmoprotection (Opu/Cho systems), mechanosensitive channels (mscL), and a robust DNA repair toolkit (recN, recG, fpg), as well as genes encoding bile salt hydrolases and heat shock proteins, providing a molecular basis for its stress resilience. These combined defense mechanisms ensure the isolates possess the necessary resilience to reach and persist in the gastrointestinal tract, a prerequisite for exerting beneficial effects in vivo.

Following the confirmation of safety and physiological resilience, the antimicrobial capacity of the isolates was subsequently evaluated. In the overlay assay, all strains inhibited the growth of *E. faecalis* ATCC 29212, *E. coli* 042, *P. aeruginosa* ATCC 27856, *S. aureus* ATCC 6538, and *S. enteritidis* ATCC 13076, with isolate M2 showing the most pronounced inhibitory effect. However, no inhibition zones were observed in the well diffusion assay using cell-free supernatants, regardless of pH conditions. Although no antimicrobial activity was detected in cell-free supernatants under the tested conditions, this does not exclude the possibility of metabolite production. Genome mining shows that M2 harbors two plantaricin genes and encodes two AMPs. This apparent discrepancy can be explained by the fact that bacteriocin expression in *L. plantarum* is often regulated by quorum-sensing and environmental stimuli, requiring specific induction conditions such as nutrient availability, oxygen levels, and stress conditions [54,55,56]. Therefore, the absence of activity in supernatants does not exclude the antimicrobial potential of these genes. Instead, it suggests that the antagonism observed in vitro was predominantly contact-dependent, while the genomic repertoire indicates latent antimicrobial capacity that could be activated under appropriate conditions, as reported for other *L. plantarum* strains [57,58,59].

Consistent with the hypothesis of direct contact-dependent mechanisms, genome analysis revealed that M2 harbors multiple adhesion-related domains (WxL, LysM, MucBP), and these bacteriocin systems may require specific induction conditions. Additionally, proteome hydropathy analysis and BATS assay revealed a predominantly hydrophilic surface, suggesting that pathogen exclusion relies on specific macromolecular interactions rather than nonspecific hydrophobic forces. Genome mining also identified two predicted antimicrobial peptides and four distinct biosynthetic gene clusters (RiPP-like, terpene, cyclic-lactone autoinducer, and type III polyketide synthase), indicating a latent antimicrobial arsenal that could be activated under specific ecological or stress conditions.

Such contact-dependent mechanisms have been previously described in certain LAB strains and may involve competitive exclusion, nutrient sequestration, or surface-associated antimicrobial factors such as adhesion proteins or exopolysaccharides [60,61,62,63,64]. In fact, genes involved in exopolysaccharides biosynthesis (GT2, GT4 families) and teichoic acid modification were abundant in the M2 genome, supporting the structural complexity of the cell envelope and its role in adhesion and biofilm formation. Microbial biofilms are physical mechanisms that enhance competitive exclusion and inhibit pathogen colonization on host epithelial surfaces [65,66]. This synergistic combination of coaggregation and biofilm formation represents a robust strategy for pathogen control in vivo [61,65].

Comparative studies have highlighted niche-specific functional and structural differences among *L. plantarum* strains. Proteomic analysis revealed that strains from vegetable matrices exhibit a more diversified surface protein repertoire (>500 proteins) compared to dairy-derived strains (~200 proteins), including adhesion-related factors such as Sortase A and bile salt hydrolase (BSH), which were detected only in vegetable isolates. These proteins, together with moonlighting enzymes like GAPDH and enolase, are implicated in mucosal adhesion and biofilm formation, supporting a mechanism of pathogen exclusion mediated by direct cell contact [67]. In contrast, a large-scale functional comparison of 107 strains from different sources (dairy, vegetable, and grape must) has demonstrated that dairy-derived strains were the most bile-tolerant and hydrophobic, while vegetable-derived strains showed superior acid resistance. Although BSH genes were widely distributed (97.2% incidence), dairy strains exhibited greater cholesterol-lowering potential, whereas vegetable strains appear better adapted to survive gastric conditions [68]. Taken together, these findings contextualize the functional profile of strain M2, its strong acid tolerance and moderate biofilm formation align with adaptations typical of plant-associated niches, while its genomic features—such as adhesion domains and CAZyme repertoire—reinforce its ecological specialization and potential for competitive exclusion rather than bile-mediated cholesterol reduction.

The probiotic potential of M2 was further supported by in vivo assays using *T. molitor* larvae, a model increasingly recognized for its ethical, economic, and biological advantages in preclinical screening. Unlike mammalian models, *T. molitor* larvae offer a rapid life cycle, low maintenance cost, and reduced ethical constraints, making them suitable for high-throughput testing of microbial safety and efficacy. In our study, larvae challenged with *E. coli* 042 and *S. enteritidis* ATCC 13076 showed significantly improved survival when treated with M2, particularly in the prophylactic regimen. Survival rates reached 70% and 100%, respectively, compared to complete mortality in untreated controls. Post-infection treatment also conferred protection, albeit to a lesser extent. Importantly, M2 alone did not induce any adverse effects, confirming its safety in a live host model. These findings provide proof of concept for the protective effects of M2 against enteric infections.

Despite the promising results, this study presents several limitations that warrant further investigation. The antimicrobial activity observed was restricted to direct contact assays, and no inhibitory effect was detected in cell-free supernatants under the tested conditions. This suggests that the mechanisms involved may be complex and context-dependent. Genome mining revealed plantaricin clusters, candidate AMPs, and unique biosynthetic gene clusters, indicating latent antimicrobial potential that could require specific induction cues. Future studies will include attempts to induce plantaricin expression through co-culture with target pathogens and stress conditions, as well as targeted extraction and characterization of surface-associated antimicrobial peptides and other cell envelope molecules using proteomic and biochemical approaches. Additionally, complementary assays—such as transcriptomic and proteomic profiling—are needed to elucidate the molecular mechanisms underlying the interaction between *L. plantarum* M2 and pathogenic bacteria. Regarding the novel BGCs identified by antiSMASH (terpene, cyclic-lactone autoinducers, and T3PKS), initial steps toward chemical characterization and heterologous expression are planned to explore their potential for producing new bioactive compounds. While the *T. molitor* model provided valuable insights into in vivo efficacy, validation in mammalian systems is essential to confirm the probiotic potential and safety of M2 in more physiologically relevant contexts.

## 5. Conclusions

Taken together, the findings of the study highlight the probiotic potential of a lactic acid bacterium isolated from the Amazonian fruit *H. balsamifera*, particularly the isolate *L. plantarum* M2. The results support its safety and adaptability to gastrointestinal-like conditions, as well as its relevance for future applications in functional foods and health interventions. Genomic and phenotypic evidence indicate traits associated with stress tolerance and competitive exclusion, positioning this isolate as a promising candidate for further development. Future research should validate its efficacy in mammalian models, investigate the functional role of antimicrobial gene clusters, and optimize cultivation conditions to induce antimicrobial compounds secretion. Assessing its scalability for clinical and industrial applications will be essential for integration into functional products and public health strategies.

## Figures and Tables

**Table 1:** Identification of Microbial Isolates Based on Morphology and MALDI-TOF MF Analysis.

Plant Species	Isolate	Gram	Catalase	MALDI-TOF MS
*A. comusus*	A1	Yeast	+	*Saccharomycodes ludwigii*
*M. zapota*	S1	Yeast	+	*Wickerhamomyces anomalus*
	S4	Yeast	+	*Wickerhamomyces anomalus*
*H. balsamifera*	M1	Gram-positive Bacillus	−	*Lactiplantibacillus plantarum*
	M2	Gram-positive Bacillus	−	*Lactiplantibacillus plantarum*
	M4	Gram-positive Bacillus	−	*Lactiplantibacillus plantarum*
	M5	Yeast	−	*Yarrowia lipolytica*
*P. insignis*	B1	Yeast	−	*Saccharomyces cerevisiae*
	B2	Yeast	+	*Pichia manshurica*
	B3	Yeast	−	*Saccharomyces cerevisiae*
	B4	Yeast	+	*Pichia manshurica*
	B5	Yeast	−	*Saccharomyces cerevisiae*
	B6	Yeast	−	*Saccharomyces cerevisiae*

+: Positive; −: Negative.

**Table 2:** Antimicrobial susceptibility of *Lactiplantibacillus plantarum* isolates.

Antibiotics	Antimicrobial Susceptibility		
	M1	M2	M4
Ampicillin	Sensitive	Sensitive	Sensitive
Ceftazidime	Sensitive	Sensitive	Sensitive
Clindamycin	Sensitive	Sensitive	Sensitive
Erythromycin	Sensitive	Sensitive	Sensitive
Gentamicin	Resistant	Resistant	Resistant
Penicillin	Sensitive	Sensitive	Sensitive
Rifampicin	Sensitive	Sensitive	Sensitive
Vancomycin	Resistant	Resistant	Resistant

**Table 3:** Comparative homology of phylogenetic marker genes between *L. plantarum* M2 and reference strains of *L. plantarum*.

Marker	Strain	Homology
16S rRNA	*Lactiplantibacillus plantarum* NBRC15891	99.66%
	*Lactiplantibacillus pentosus* 124-2	99.54%
	*Lactiplantibacillus plantarum* NRRL B-14768	99.53%
	*Lactiplantibacillus plantarum* JCM1149	99.52%
	*Lactiplantibacillus plantarum* CIP103151	99.48%
*gyrB*	*Lactiplantibacillus plantarum* LRLD-22	100%
	*Lactiplantibacillus plantarum* DSM20174	100%
	*Lactiplantibacillus plantarum* TMW 1.1623	100%
	*Lactiplantibacillus plantarum* CNEI-KCA5	100%
	*Lactiplantibacillus plantarum* TMW 1.277	100%
*gyrA*	*Lactiplantibacillus plantarum* TMW 1.1478	100%
	*Lactiplantibacillus plantarum* L55	99.96%
	*Lactiplantibacillus plantarum* N3	99.96%
	*Lactiplantibacillus plantarum* FBL-3A	99.96%
	*Lactiplantibacillus plantarum* CACC558	99.96%
23S rRNA	*Lactiplantibacillus plantarum* MJ726	99.93%
	*Lactiplantibacillus plantarum* LL441	99.93%
	*Lactiplantibacillus plantarum* DS1073	99.93%
	*Lactiplantibacillus plantarum* NBC99	99.93%
	*Lactiplantibacillus plantarum* LRLD-22	99.93%
*recA*	*Lactiplantibacillus plantarum* MJ726	100%
	*Lactiplantibacillus plantarum* LRLD-22	100%
	*Lactiplantibacillus plantarum* WHH1701	100%
	*Lactiplantibacillus plantarum* TMW 1.1623	100%
	*Lactiplantibacillus plantarum* JBE245	100%

**Table 4:** Aggregation and surface properties of *Lactiplantibacillus plantarum* M2.

Parameter	Value (%)
Autoaggregation	7.8 ± 2.1
Coaggregation with *E. coli*	7.0 ± 3.0
Coaggregation with *S. enterica*	21.1 ± 7.3
Coaggregation with *E. faecalis*	20.2 ± 0.4
Affinity to toluene	−4.75 ± 0.46
Affinity to chloroform	2.16 to 1.14
Affinity to ethyl acetate	2.61 ± 0.99

**Table 5:** Biosynthetic Gene Clusters (BGCs) identified in the *Lactiplantibacillus plantarum* M2 genome.

Cluster Type	Size (bp)	Similarity to Known Cluster (MIBiG)
RiPP-like	12,151	—
Terpene	12,414	—
Cyclic-lactone autoinducer	20,706	—
T3PKS	26,017	—

## Data Availability

The raw data supporting the conclusions of this article will be made available by the authors on request.

## Supplementary Materials

The following supporting information can be downloaded at: https://www.mdpi.com/article/10.3390/pharmaceutics17121557/s1, Table S1: CLSI interpretive criteria for classification of antimicrobial susceptibility.
