# Supplementary material for: Probiotic Potential and Genome-Based Characterization of Lactiplantibacillus plantarum M2, a Promising Isolate Obtained from Spontaneous Fermentation of Humiria balsamifera Pulp
Source: Pharmaceutics. 2025 Dec 3;17(12):1557. doi: 10.3390/pharmaceutics17121557 (PMC12736787; doi:10.3390/pharmaceutics17121557)
Supplement: Supplementary file 1 [file pharmaceutics-17-01557-s001.zip › pharmaceutics-4000116-supplementary.pdf]

Table S1. CLSI interpretive criteria for classification of antimicrobial susceptibility.

**Antibiotics**	**Disc content (μg)**	**CLSI interpretive criteria**		
		**Resistant**	**Intermediate**	**Sensitive**
Ampicillin	10 μg	≤ 13	14-16	≥ 17
Ceftazidime	30 μg	≤ 17	18-20	≥ 21
Clindamycin	02 μg	≤ 15	16-18	≥ 19
Erythromycin	15 μg	≤ 13	14-22	≥ 23
Gentamicin	10 μg	≤ 12	13-14	≥ 15
Penicillin	10 μg	≤ 14	-	≥ 15
Rifampicin	05 μg	≤ 16	17-19	≥ 20
Vancomycin	30 μg	≤ 14	15 – 16	≥ 17

R = resistant; I = intermediate; S = sensitive;
